# Long non-coding RNAs in brain tumors: roles and potential as therapeutic targets

**DOI:** 10.1186/s13045-021-01088-0

**Published:** 2021-05-12

**Authors:** Sung-Hyun Kim, Key-Hwan Lim, Sumin Yang, Jae-Yeol Joo

**Affiliations:** Neurodegenerative Disease Research Group, Korea Brain Research Institute, Daegu, 41062 Republic of Korea

**Keywords:** Brain disease, Genomics, Glioma, LncRNA, Neurodegenerative disease

## Abstract

Brain tumors are associated with adverse outcomes despite improvements in radiation therapy, chemotherapy, and photodynamic therapy. However, treatment approaches are evolving, and new biological phenomena are being explored to identify the appropriate treatment of brain tumors. Long non-coding RNAs (lncRNAs), a type of non-coding RNA longer than 200 nucleotides, regulate gene expression at the transcriptional, post-transcriptional, and epigenetic levels and are involved in a variety of biological functions. Recent studies on lncRNAs have revealed their aberrant expression in various cancers, with distinct expression patterns associated with their instrumental roles in cancer. Abnormal expression of lncRNAs has also been identified in brain tumors. Here, we review the potential roles of lncRNAs and their biological functions in the context of brain tumors. We also summarize the current understanding of the molecular mechanisms and signaling pathways related to lncRNAs that may guide clinical trials for brain tumor therapy.

## Background

**Fig. 1:**
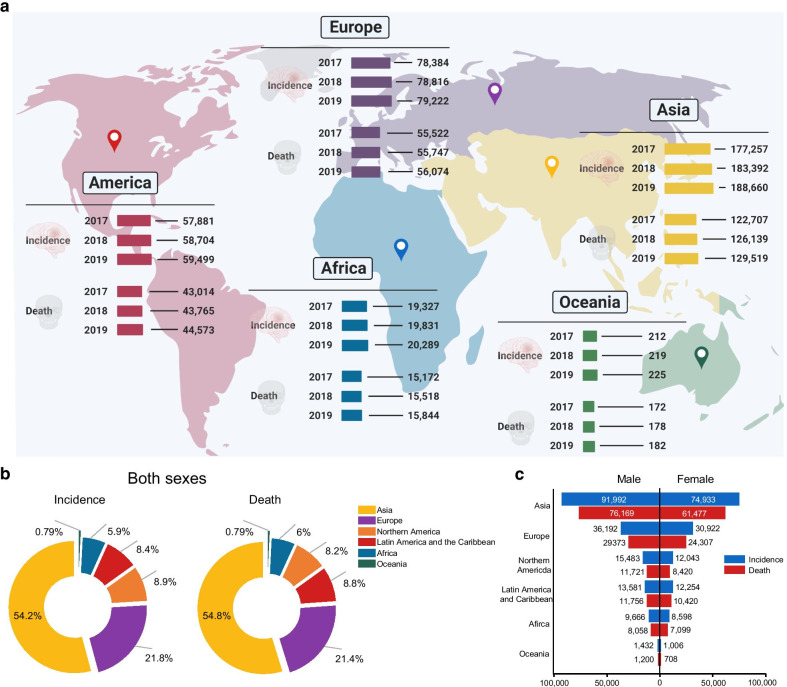
Global trends of brain tumor incidence and death. **a** Global map showing the incidence and deaths related to brain tumor across the world from 2017 to 2019. **b** Pie charts present the distribution of incidence and deaths related to brain tumor in major world regions in 2020 for both sexes and all ages. **c** Bar graph reveals the incidence of brain tumor and corresponding deaths in 2020 in males and females. The data sources and methods used for each world region and detailed condition of the estimates are available online from global health data exchange and GLOBALCAN 2020 at the Global Cancer Observatory (GCO)

The occurrence of brain tumors is increasing worldwide and is more common among men than women [1] (Fig. 1). Glioma, a heterogeneous group of tumors, is one of the most common and major types of malignant brain tumors of the central nervous system. The World Health Organization has stated that according to cellular origin and based on histological subtypes and genetic features, gliomas can be subclassified into astrocytoma, oligoastrocytoma, oligodendroglioma, or glioblastoma. Moreover, each class of gliomas is categorized into grades I, II, III, and IV to assess the degree of malignancy, where grade I is the least malignant and grade IV is the most malignant [2, 3]. In addition, brain tumors afflict a large number of individuals worldwide and are difficult to detect and treat. One problem associated with the treatment of such tumors is the blood–brain barrier, a specific system of endothelial cells that prevents molecules in the circulating blood from non-selectively crossing into the extracellular fluid of the central nervous system to protect the neural cells and preserve neural homeostasis, which makes drug diffusion inefficient or impossible. Although there have been improvements in surgical and other therapeutic approaches, non-specificity of the potentially toxic drugs used to treat the condition remains an issue [4, 5]. Therefore, alternative approaches to treatment of brain tumors targeting different kinds of molecules are actively being pursued.

**Fig. 2:**
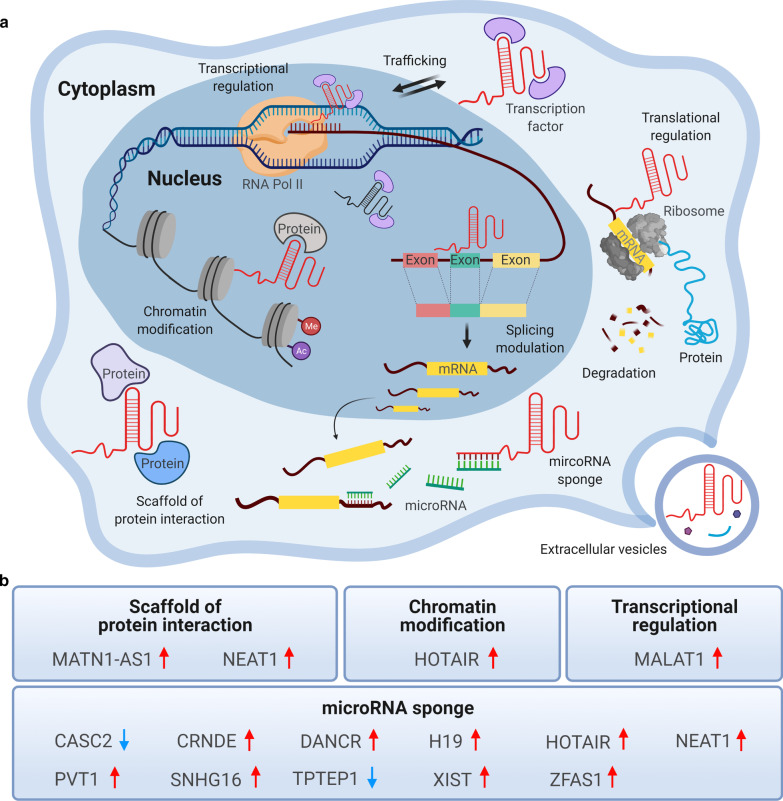
Classification of lncRNAs based on their functional mechanisms. **a** The multiple functional roles of lncRNAs. In the nucleus, lncRNAs are involved in transcription regulation (activation or repression) via acting as transcription factors, splicing of mRNA, and chromatin modification. In the cytoplasm, they regulate translation by interacting with ribosomes, sponging miRNAs, degrading mRNAs, scaffolding protein interactions, and trafficking transcription factors. Moreover, lncRNAs can be enclosed and released by extracellular vesicles, such as exosomes. **b** The functional contributions and expression levels of reported lncRNAs in brain tumor

Previously, non-coding regions of the genome, which do not translate into proteins, were regarded as junk DNA. However, high-throughput technologies have accelerated and improved our understanding of the non-coding genome. Studies have revealed that long non-coding RNAs (lncRNAs), whose transcripts are longer than 200 nucleotides, are involved in a wide range of biological processes [6] (Fig. 2). lncRNAs regulate not only gene expression but also epigenetic, transcriptional, and post-transcriptional modification, and their expression may be closely related to diverse biological functions including cell survival [7–9]. In the past two decades, various roles of lncRNAs have been implicated in tumor biology [10]. The dysregulation of lncRNAs is increasingly being associated with many human diseases, especially cancers. lncRNAs exhibit unique expression patterns in various types of cancers, and play instrumental roles as cancer suppressors or promoters. Additionally, aberrant lncRNA expression in cancers has been linked to modulated expression of genes involved in tumorigenesis, metastasis, and tumor stage progression [11–16]. Moreover, several studies have revealed that lncRNAs control post-translational modification by functioning as molecular sponges or competing endogenous RNAs (ceRNAs) that modulate microRNAs (miRNAs), which in turn regulate the expression of target genes [17–19]. Furthermore, lncRNAs are involved in signal transduction pathways, miRNA silencing, DNA damage, cell cycle control, epigenetic regulation, and hormone-driven disease states, all of which play key roles in cancer progression and metastasis [20].

Recent studies have shown that aberrantly expressed lncRNAs are closely involved with the pathology and prognosis of gliomas. These studies also identified the molecular mechanisms underlying lncRNA activity, which eventually modulate biological processes in brain tumors [21]. In addition to these molecular insights, advances in genome-wide association technologies have led to the discovery of several types of lncRNAs in brain cancer. Here, we review the current understanding of the functions of lncRNAs in brain tumors by discussing latest findings from diverse mammalian systems.

## LncRNAs in signaling pathways and brain tumors

**Fig. 3:**
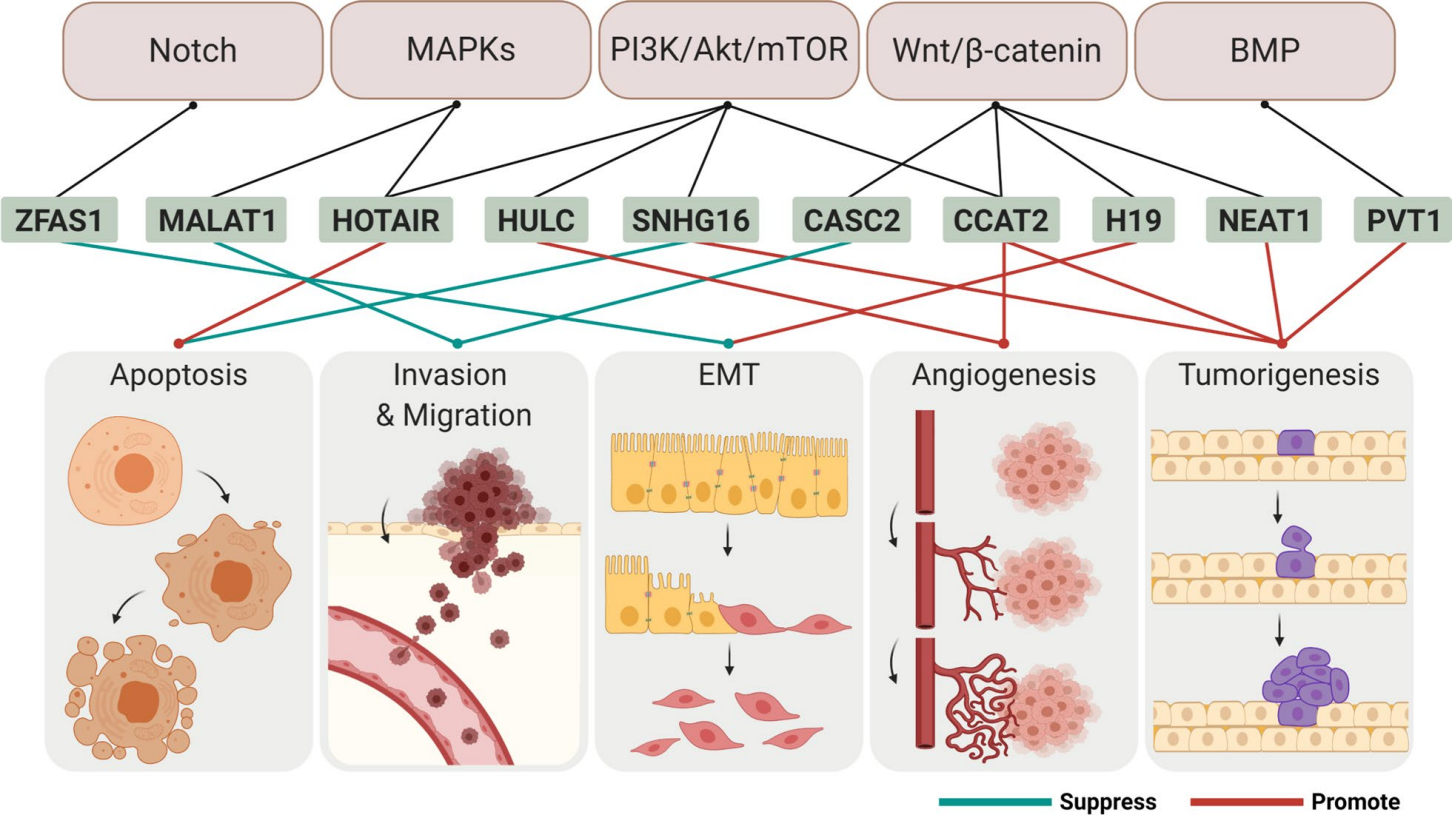
LncRNAs regulate cellular processes in the tumor cell through diverse signaling pathways. Based on the genetic characteristics and biochemical studies of lncRNAs, it has been proposed that lncRNAs are required for the coordination of several intracellular pathways, such as PI3K-Akt and Wnt pathways. lncRNAs are generated by transcriptional processes when particular signaling pathways are activated. Moreover, expression of lncRNAs is linked to physiological processes of tumor cells

The roles of lncRNAs and their associated pathways in gliomas have been identified, and their interactions with miRNAs have been studied [21, 22]. This section describes the mechanisms of action of lncRNAs in cellular processes and signaling pathways involved in the development of gliomas (Fig. 3).

### PI3K/Akt/mTOR pathway

The PI3K/Akt/mTOR pathway is often deregulated in cancer cells, leading to enhanced cell growth, proliferation, and survival of these cells. This pathway also plays a crucial role in apoptosis and autophagy in glioma [23, 24].

The cancer susceptibility candidate 2 (CASC2) lncRNA, which is downregulated gene in various cancers including gliomas, is an important modulator of this pathway. CASC2 has been identified as a tumor suppressor that is located on chromosome 10q26 [25, 26]. CASC2 modulates glioma growth and resistance to temozolomide (TMZ), one of the most commonly used chemotherapeutic agents for glioma, by upregulating the expression of phosphatase and tensin homolog (PTEN), a known tumor suppressor involving in several cancers including gliomas and inhibits Akt signaling pathway and p-Akt pathway via direct inhibition of miR‐181a [27]. Moreover, it negatively regulates miR-193a-5p expression, which in turn reduces TMZ-induced autophagy via upregulation of the mTOR pathway [28].

The differentiation antagonizing non-protein coding RNA (DANCR) lncRNA, a tumor-associated lncRNA located on chromosome 14, is upregulated in several types of human cancers [29]. Aberrant expression of DANCR in various cancers is associated with oncogenic or tumor suppressive mechanisms that are hallmarks of cancer, such as cell proliferation, invasion, and metastasis [29–31]. Additionally, DANCR upregulates AXL, a receptor tyrosine kinase that activates the PI3K/Akt/NF-κB signaling pathway [32, 33] by competitively binding with miR-33a-5p, miRNA-33b-5p, miR-1-3p, miR-206 and miR-613 to mediate the cisplatin resistance in gliomas [34].

The highly upregulated in liver cancer (HULC) lncRNA is located on chromosome 6p24.3 and highly expressed in glioma as an oncogene that contributes to tumor progression [35]. HULC silencing suppresses proliferation, adhesion, migration, invasion, and angiogenesis through the regulation of the cell cycle and anoikis, a Greek word for "homelessness" and a critical mechanism in preventing tumor cell apoptosis via a decrease in cell–matrix interaction, in the glioma by modulating endothelial cell-specific molecule 1 (ESM-1) expression via the PI3K/Akt/mTOR signaling pathway [36].

The small nucleolar RNA host gene 16 (SNHG16) lncRNA is highly expressed in gliomas and is correlated with short survival and poor clinicopathologic features. Knockdown of SNHG16 suppresses the viability of glioma cells and promotes apoptosis by regulating the expression of B-cell lymphoma 2 family (Bcl-2) proteins and activating the PI3K/Akt signaling pathway. Moreover, SNHG16 promotes glioma tumorigenesis by acting as the ceRNA that upregulates the expression of the miR-4518 targeted gene, protein arginine methyltransferase 5 (PRMT5) by directly sponging miR-4518 [37]. Additionally, high expression of SNHG16 increases cell migration, invasion, and proliferation via the PI3K/Akt pathway and sponging of miR-373, leading to release of epidermal growth factor receptor (EGFR), which is an important receptor tyrosine kinase in tumorigenesis and a target of miR-373. Thus, SNHG16 enhances the tumorigenicity of gliomas through the miR-373/EGFR axis via the PI3K/Akt pathway [38, 39].

The colorectal neoplasia differentially expressed (CRNDE) lncRNA, which is specifically expressed in certain regions of the human brain, is also upregulated in gliomas [40]. This lncRNA negatively regulates miR-136-5p by binding as a ceRNA, thereby inhibiting Bcl-2 and Wnt2, which act downstream of the PI3K/Akt/mTOR signaling pathway, thus enhancing the glioma malignancy [41]. Additionally, CRNDE modulates the mTOR signaling pathway; knockdown of the CRNDE decreases the phosphorylation of P70S6K, a downstream signaling molecule in the mTOR signaling pathway [42].

The HOX transcript antisense RNA (HOTAIR) lncRNA is a ~ 2.2 kb lncRNA belonging to the homeobox superfamily that is transcribed from the HOXC locus at chromosome 12q13. HOTAIR acts as a negative prognostic factor for various cancers, including glioma, and its abundant expression is closely related to tumor progression [43, 44]. Knockdown of HOTAIR inhibits fibroblast growth factor 1 (FGF1) expression and blocks the activity of the PI3K/Akt and MEK1/2 pathways, leading to the suppression of malignant behavior, by upregulating miR-326, which targets FGF1. This intervention induces cell cycle arrest and apoptosis, and inhibits cell migration, invasion, and proliferation in human glioma cells [45].

The X-inactive-specific transcript (XIST) lncRNA, produced by the XIST gene, is a critical regulator of cancer, levels of which correlate with cell differentiation, proliferation, and genome maintenance. Abnormal expression of XIST has been seen in various human cancers, where it plays a pathological function [46, 47]. Moreover, XIST expression levels are increased in glioma and human glioma stem cells. Attenuation of cell growth and glucose metabolism was seen in glioblastoma cells following knockdown of XIST. This effect occurs via regulation of the insulin receptor substrate 1 (IRS1)/PI3K/Akt pathway as XIST functions as the ceRNA of miR-126; in vivo, this effect reduced glioblastoma tumor growth [48].

The cancer susceptibility candidate 2 (CCAT2) lncRNA is an oncogenic 1,752 bp lncRNA located on chromosome 8q24.21 that is upregulated in various human cancers, including glioma [49]. CCAT2 enhances cell proliferation and endothelial angiogenesis in glioma by increasing vascular endothelial growth factor A (VEGFA) through miR-424 and activating the PI3K/Akt signaling pathway[50].

### MAPK pathway

Mitogen-activated protein kinases (MAPKs) are a family of signaling molecules, which are activated by growth factors, cytokines, and environmental stress. They control crucial cellular processes, including cell growth, proliferation, migration, and apoptosis [51].

The metastasis-associated lung adenocarcinoma transcript 1 (MALAT1) lncRNA, also called nuclear-enriched transcript 2, is an 8.7 kb lncRNA located on chromosome 11q13. MALAT1 is known as a prognostic indicator in non-small cell lung cancer, but is abnormally expressed in various human cancers; it is upregulated in gliomas, where its expression is associated with the progression of glioma and poor prognosis [52–54]. Additionally, the tumor-suppressive function of MALAT1 is related to inhibition of the extracellular signal-regulated kinase/mitogen-activated protein kinase (ERK/MAPK) signaling pathway, which modulates cell proliferation and invasion, as well as inhibition of matrix metalloproteinase 2 (MMP2), a key enzyme in invasion. Therefore, knockdown of MALAT1 promotes cell proliferation and invasion in glioma [55]. Similarly, downregulation of MALAT1 enhances cell proliferation via activation of the MAPK pathway in glioma stem cells [56].

The downregulation of MATN1 antisense RNA 1 (MATN-AS1) lncRNA is associated with short survival duration and poor prognosis of glioblastoma patients. Upregulation of MATN1-AS2 suppressed the expression of RELA, a group of the Rel proteins family and MAPK pathway, thereby inhibiting cell proliferation and invasion through apoptosis of glioblastoma cells [57].

The transmembrane phosphatase with tensin homology pseudogene 1 (TPTEP1) lncRNA is one of the most downregulated lncRNAs in non-small cell lung cancer, where it inhibits the cell proliferation by competitively sponging miR-328-5p [58]. In glioma, TPTEP1 promotes the P38 MAPK pathway through competitive interactions with miR-106a-5p, thus inhibiting stemness and radioresistance [59].

### Wnt/β-catenin pathway

Wnt/β-catenin signaling is essential in embryonic development and tissue homeostasis, and thus controls cell proliferation and cell fate. Hence, activation and mutation of the Wnt/ β-catenin pathway is associated with various human cancers and other diseases [60, 61].

Overexpression of CASC2 significantly suppresses migration, invasion, and proliferation in glioma through the inhibition of Wnt/β-catenin signaling pathway [62].

DANCR, which is upregulated in glioma, that activates the Wnt/β-catenin signaling pathway, leading to increased migration and proliferation of glioma cells. However, this tendency is reduced by si-DANCR [63].

The H19 lncRNA is located downstream of insulin growth factor 2 (IGF2) on chromosome 11p15 in humans and chromosome 7 in mice. Its expression is increased in various cancers including gliomas, where it correlates with cell growth and invasion and it plays an oncogenic role [64–67]. Furthermore, H19 expression is closely related with glioma grade [68]. Conversely, downregulation of H19 suppresses glioma cell progression, including, migration, invasion, and proliferation via suppressing the Wnt/β-catenin signaling pathway [69]. Silencing of H19 suppresses epithelial–mesenchymal transition (EMT), which induces cell migration and decrease intercellular adhesion, and is associated with chemoresistance in various cancers, thus, decreasing chemoresistance to TMZ via suppressing the Wnt/β-catenin signaling pathway [70].

The nuclear-enriched abundant transcript 1 (NEAT1) lncRNA, a 3.7 kb lncRNA found on chromosome 11q13.1, localizes to the nucleus and is an essential core component of the paraspeckle substructure [71, 72]. This lncRNA is upregulated in some cancers, including glioma, where it plays as an oncogene, and is involved in proliferation, migration, invasion, and impaired apoptosis [73–75]. Moreover, an increase in this lncRNA enhances progression of glioblastoma and tumorigenesis via the effects of the EGFR pathway on cell cycle regulation. This involves binding of enhancer of zeste 2 (EZH2) and the Wnt/β-catenin signaling pathway [76].

Wnt inhibitory factor 1 (WIF1) attenuates non-canonical Wnt signaling through downregulation of MALAT1, thereby suppressing migration, but not proliferation, of glioblastoma cells [77].

Similarly, knockdown of CCAT2 suppresses cell migration, proliferation, cell cycle progression, and tumorigenesis in glioma by downregulating the Wnt/β-catenin signaling pathway [78].

### Other pathways

In addition to these pathways, several other pathways, including the miRNA axis, bone morphogenetic protein (BMP), Notch, nuclear factor kappa-light-chain-enhancer of activation B cells (NF-κB) pathways are involved in cancer. miRNAs are small non-coding RNAs that are 21–25 nucleotides in length, which mainly regulate expression of gene at the post-transcriptional level, and the expression of protein via binding to their target mRNAs [79]. BMP protein is a part of the transforming growth factor-β (TGF-β) family and plays pivotal roles in the morphogenesis of different organs, including the neural system. BMP signaling has also been reported as a tumor promoter as well as a tumor suppressor in various types of cancer [80]. Notch signaling is a conserved ligand-receptor signaling pathway that is involved in fundamental mechanistic functions, such as cell survival, differentiation, development, and apoptosis. Dysregulation of Notch signaling has been reported in various diseases including cancer [81]. Finally, NF-κB is one of the long-suspected factors that mediates interactions between cancer and inflammation. NF-κB signaling pathway regulates cell migration, proliferation, inflammation, and apoptosis by modulating the expression of anti-apoptotic molecules and pro-inflammatory factors [82].

The overexpression of plasmacytoma variant translocation 1 (PVT1) lncRNA located on chromosome 8q24, has been reported to play an important role in tumor progression and biological processes, such as apoptosis, invasion, mobility, and proliferation in various types of cancer [83–85]. It affects the progression of glioma by targeting miRNAs, such as miR-186 [86], miR-200a [87], miR-424 [88], and miR-140-5p [89]. Knockdown of this lncRNA suppresses malignant behaviors in glioma through the binding and upregulation of miR-190a-5p and miR-488-3p, which target and inhibit myocyte enhancer factor 2C (MEF2C), which is also transcriptionally regulated by the oncogene jagged 1 (JAG1) [90]. Furthermore, this lncRNA modulates Gremlin 1 (GREM1) and BMP downstream signaling pathway through sponging of miR-128-3p, thereby promoting tumorigenesis and progression in gliomas [91].

As mentioned previously, CASC2, which is downregulated in glioma, modulates miRNAs. Overexpression of CASC2 inhibits glioma malignancy, migration, invasion, and proliferation and induces cell apoptosis via negative modulation of miR-21 [92].

The ZNFX1 antisense RNA 1 (ZFAS1) lncRNA located on chromosome 20q13 is another lncRNA involved in progression and metastasis of various cancers [93]. ZFAS1 induces glioma progression by sponging miR‐150‐5p to regulate proteolipid protein 2 (PLP2), which is known as the functional target of miR‐150‐5p [94]. Inhibition of ZFAS1 significantly suppresses proliferation, migration, and invasion of glioma cells. Furthermore, ZFAS1 knockdown reduces the viability of glioma cells and increases their cisplatin chemosensitivity by targeting miR-432-5p [95]. Moreover, the EMT and the Notch signaling pathway are inactivated in the glioma cells after ZFAS1 knockdown. Thus, ZFAS1 exhibits an oncogenic role in glioma progression by regulating the EMT and the Notch signaling pathway [96].

Similarly, DANCR promotes glioma progression by acting as a sponge of miR-634, thus modulating RAB1A, which has an important role in tumor progression [97]. Furthermore, knockdown of DANCR revealed its role as the ceRNA that regulates expression of B-lymphoma Moloney murine leukemia virus insertion region-1 (BMI1), a self-renewal gene that is associated with tumorigenesis and progression of cancer in glioma [98]. Thus, knockdown of DANCR inhibits migration, invasion, and proliferation of glioma cells via sponging of miR-135a-5p [99].

The ceRNA role of SNHG16, which is overexpressed in glioma, also promotes the malignancy through the enhancement of endogenous target gene, E2F1 via sponging of miR-20a-5p [100].

Knockdown of CRNDE promotes the miR-348 expression, leading to inhibition of piwi-like RNA-mediated gene silencing 4 (PIWIL4), which has an oncogenic role in glioma, thus inhibiting the malignant progression of glioma [101]. CRNDE also downregulates miR-186, which is known to have a malignant biological role in glioma stem cells [102].

Glioma malignancy is also promoted by HOTAIR via its interactions with the polycomb repressive complex 2 (PRC2) and certain miRNAs [103]. Also, HOTAIR expression correlates with angiogenesis via modulation of VEGFA expression, one of the most important factors in tumor angiogenesis, and transmission of this factor to endothelial cells through glioma cell-derived extracellular vesicles [104]. HOTAIR also regulates glutaminase levels to enhance glioma progression via binding of miR-126-5p as a ceRNA [105]. High expression of HOTAIR was found to interact with miR-148b-3p and inhibit malignancy in glioma cell [106]. HOTAIR regulates the 3’ untranslated region of spindle and kinetochore associated complex subunit 2 (SKA2), and binds the tumor suppressor miR-141 as an endogenous sponge, thus participating in the pathogenesis and progression of human gliomas [107]. Knockdown of HOTAIR increased the permeability of the blood–tumor barrier (BTB) by connecting with miR-148b-3p as a target RNA. This knockdown also decreased the levels of tight junction-correlated proteins, such as Zonula occludens 1 (ZO-1), occludin, and claudin-5, in glioma microvascular endothelial cells by targeting upstream stimulatory factor 1 (USF1), thus enabling drug delivery [108]. Finally, HOTAIR is regulated by the glioblastoma proliferative pathway, specifically by bromodomain and extra-terminal domain proteins, such as bromodomain-containing 4 (BRD4), which binds to and increases HOTAIR levels in this pathway [109].

The H19 lncRNA increases proliferation and invasion via downregulating miR-152 [110], and miR-140, which targets iASPP [111] and driving of the miR-675 [66, 68, 112, 113]. It also regulates glioma angiogenesis by inhibiting miR-29a, which targets and represses the angiogenic factor vasohibin 2 (VASH2) [114]. H19 acts as a ceRNA on miR-138, which targets HIF-1α and modulates the expression of VEGF, thus promoting proliferation, migration, invasion, and angiogenesis in gliomas [115]. In addition, H19 regulates levels of the sex determining region Y-box protein 4 (SOX4) by sponging miR-130a-3p as a ceRNA. These findings indicate that this lncRNA regulates the EMT process, in which epithelial cells lose their original roles and gain migratory and invasion-related roles, becoming mesenchymal cells, as seen in various tumors [116]. H19 stimulation via oxidative stress promotes TMZ resistance through activation of the NF-κB signaling pathway [117].

Similarly, XIST regulates miR-429, thus promoting tumorigenicity and angiogenesis in gliomas [118]. XIST is correlated with miR-29c and modulates TMZ chemoresistance via its effects on DNA mismatch repair-related molecules, such as O^6^-methylguanine-DNA methyltransferase (MGMT) and specificity protein 1 (SP1) [119]. XIST also has oncogenic functions in glioma; it inhibits miR-137 by acting as a sponge, thereby increasing Ras-related C3 botulinum toxin substrate 1(Rac1) expression [120]. Knockdown of XIST upregulates miR-137, enhancing BTB permeability and decreasing angiogenesis in glioma via inhibition of the transcription factors forkhead box C1 (FOXC1) and ZO-2 [121]. It also upregulates miR-152, suppressing cell cycle progression and increasing apoptosis in glioma stem cells, thus playing a tumor suppressive role [122].

NEAT1 directly targets miR-449b-5p as a molecular sponge and promotes upregulation of c-Met, a direct target of miR-449b-5p, to increase pathogenesis in glioma [123]. Meanwhile, knockdown of NEAT1 activates miR-let-7e, which acts as a cancer inhibitor by decreasing migration, invasion, and proliferation while increasing apoptosis in glioma stem cells and this leads to downregulation of NRAS protein, an isoform of RAS reported to be mutated in several cancers and known to play an oncogenic role in glioma stem cells, thus inhibiting disease progression [124]. Similarly, NEAT1 knockdown targets miR-132 [125], which regulates the oncogene sex determining region Y-box protein 2 (SOX2) and miR-107 [126, 127], a modulator of cyclin-dependent kinase 6 (CDK6) and CDK14; these findings were closely connected with glioma malignancy. NEAT1 knockdown also promotes BTB permeability as it binds to miR-181-5p and targets sex determining region Y-box protein 5 (SOX5). Thus, knockdown of this lncRNA reduces expression of tight junction proteins, such as ZO-1, occludin, and claudin-5 in glioma microvascular endothelial cells [128].

MALAT1 increases the level of zinc fingers and homeoboxes 1 (ZHX1), which plays a role in the carcinogenesis of several cancers including glioblastoma by acting as a transcription repressor via molecular sponging of miR-199a and as a ceRNA, thereby promoting proliferation and progression of glioblastoma [129]. MALAT1 also acts as a tumor promoter, enhancing the tumorigenesis of glioma stem cells via modulation of miR-129 and subsequent suppression of SOX2 [130]. In addition, MALAT1 enhances progression of glioma cells via upregulation of Rap1B, STMN1, RAB5A, and ATG4D by sponging miR-101 [131, 132]. Moreover, MALAT1 promotes chemoresistance to TMZ in glioblastoma cells by repressing miR-101[133] and miR-203 [134]. However, MALAT1 also plays tumor-suppressive roles that involve repression of miR-155 and enhancement of F-box and WD repeat domain-containing 7 (FBXW7) function, which downregulates the expression of numerous oncoproteins in glioma cells [135].

In glioma, MATN1-AS1 enhances the carcinogenesis by sponging the miR-200b/c/429 as the ceRNA to regulate chromodomain helicase DNA-binding protein 1 (CHD1), which has an important role in the determination of cell fate [136].

CCAT2 also acts as a molecular sponge to downregulate miR-424 and upregulates Chk1, thereby enhancing chemotolerance in glioma cells [137].

## LncRNAs and brain tumor immunity

The immune system shows responses to pathogens that may be categorized as part of innate or adaptive immunity. Pathogen-infected cells synthesize inflammatory mediators and cytokines via transcriptional and post-transcriptional regulation of genes as part of the immune response. Such immune responses start with discriminative recognition of pathogens by certain receptors, such as toll-like receptors [138]. Toll-like receptors recognize ligands and stimulate synthesis of inflammatory factors and cytokines through transcriptional regulation of genes via transcription factors, such as NF-κB and post-transcriptional regulation of gene expression through miRNAs [138, 139]. These miRNAs regulate the activation of T cells and the release of inflammatory cytokines, such as tumor necrosis factor α, interleukin, and interferon β [140–142]. Additionally, lncRNAs modulate the expression of immunogenes through transcriptional regulation [143]. LncRNAs also play various roles during an immune reaction including immune activation, immune cell migration, infiltration, antigen presentation, and antigen release as part of the immune response against cancer [144, 145]. Therefore, miRNAs and lncRNAs are important regulators involved in the activation of immunocytes during the pathology of tumor [146].

In anaplastic glioma patients, nine immune-related lncRNAs (SNHG8, PGM5-AS1, ST20-AS1, LINC00937, AGAP2-AS1, MIR155HG, TUG1, MAPKAPK5-AS1, and HCG18) have been identified. Among these, PGM5-AS1, ST20-AS1, AGAP2-AS1 and MIR155HG have been identified as risk-related genes, whereas SNHG8, LINC00937, TUGI, MAPKAPK5-AS1 and HCG18 are considered protective genes. The expression of these genes reveals differences in immune status among low- and high-risk groups. These immune-related lncRNAs directly or indirectly regulate several prognostic immune-related mRNAs [147]. Similarly, 11 immune-related lncRNA signatures have been identified in glioma patients: H19, DLGAP1.AS1, AC025171.1, PAXIP1.AS2, FEZF1.AS1, HOTAIRM1, HOXD.AS2, and ARHGEF26.AS1 are upregulated and LINC00205, WDR11.AS1, and THAP7.AS1 are downregulated in a high-risk glioma group compared to a low-risk group [148, 149]. A recent study reported four prognostic lncRNA (AK098425, AL833059, AK056155 and CR613436) that are coexpressed with protein-coding genes and are significantly associated with immune responses, such as the inflammatory response, innate immune response, and B cell-mediated immunity in glioblastomas [150]. Similarly, six immune-related lncRNA (AC005013.5, UBE2R2-AS1, ENTPD1-AS1, RP11-89C21.2, AC073115.6, and XLOC-004803) were found to be closely associated with immune-related biological processes and pathways in glioblastomas [151]. In addition, the lncRNA HOTAIR myeloid-specific 1 (HOTAIRM1), whose expression is upregulated in glioma, enhances malignancy via sponging of miR-129-5p as ceRNA and is also related to immune activation that promotes T cell-mediated immune responses [152]. Thus, these lncRNAs are useful for the development of specific immunotherapies for glioma patients. Targeting the immune response with cancer therapeutics is a promising new strategy for the treatment of gliomas.

## Exocytosis and endocytosis of lncRNA in brain tumors

Extracellular vesicles (EVs) are nano-sized membranous and lipid bilayer vesicles. They can be divided into exosomes, ectosomes, microvesicles, and apoptotic bodies based on their biogenesis and size [153]. These vesicles are crucial factors in various disease processes, including in neurodegenerative diseases [154]. Exosomes ranges from 30 to 100 nm in diameter and are generated through the endosomal pathway by the exocytosis of multivesicular bodies from the plasma membrane. Exosomes are released by various types of cells, such as normal, immune, and tumor cells [155]. Released exosomes can be enclosed by neighboring cells or by recipient cells [156, 157]. Moreover, exosomes can transport various types of molecules, such as proteins, mRNAs and lncRNA that play important roles in cell to cell communications. Such exchange of genetic and epigenetic information through intercellular communication promotes epigenetic changes in cells of different tissues [158]. Interestingly, lncRNAs are selectively sorted into exosomes and the released exosomal lncRNAs can reveal differences in expression levels between normal cells and cancer cells. Hence, cancer cells show discrepancies in expression of specific exosomal lncRNAs, which may be involved in various functions [159, 160]. Several recent studies have reported the multiple functions of exosomal lncRNAs in cancer, including in tumorigenesis, angiogenesis, drug resistance, and regulation of tumor microenvironment [154, 161–163].

The POU class 3 homeobox 3 (POU3F3) lncRNA is an oncogene seen in various human cancers, such as cervical cancer, nasopharyngeal carcinoma, and breast cancer [164]. In glioma, expression of this lncRNA is upregulated and enriched exosomes released by glioma cells enhance angiogenesis [165].

Similarly, the SBF2 antisense RNA 1 (SBF2-AS1) lncRNA, located on the 11p15.1 locus, has been identified as a critical regulation factor in cancer progression [166, 167]. In glioblastoma, SBF2-AS1 can be transferred into exosomes and secretion of such oncogenic SBF2-AS1-enriched exosomes promotes chemoresistance to TMZ. SBF2-AS1 acts as a ceRNA molecular sponge of miR-151a-3p, thus promoting oncogenesis [168].

CCAT2-enriched exosomes are also released by glioma cells and may also be involved in human umbilical vein endothelial cells. In these endothelial cells, upregulation of CCAT2 enhances angiogenesis via increasing vascular endothelial growth factor A and transforming growth factor β expression and inhibits apoptosis via increasing Bcl-2 and decreasing Bcl-2-associated X (Bax) and caspase-3 expression. Therefore, the release of exosomes containing the lncRNA CCAT2 by glioma cells promotes angiogenesis and inhibits endothelial cell apoptosis [169].

LncRNA activated by transforming growth factor (TGF)-β (lncRNA-ATB), is abnormally expressed and functions as a progression factor in various cancers, such as hepatocellular carcinoma, gastric cancer, and breast cancer [170]. This exosomal lncRNA is upregulated in glioma and activates astrocytes, but connects and suppresses miR-204-3p in astrocytes. Thus, this lncRNA enhances the invasion of glioma cells [171].

## Conclusions and perspectives

In 1955, Georges Palade, the cell biologist, identified the first ncRNA, which was linked to the ribonucleotide protein complex. This was followed by discoveries of regulatory ncRNAs, such as miRNAs, ribosomal RNAs, transfer RNAs, small nuclear RNAs, short interfering RNAs, ceRNAs, enhancer RNAs, piwi-interacting RNAs, and lncRNAs in the both prokaryotic and eukaryotic organisms [172]. However, ncRNAs were considered as ‘junk’ products of gene transcription processes and study of the ncRNAs was hampered by the lack of genetic information in the past decades. However, the roles of ncRNAs have been gradually elucidated to understand the genetic heterogeneity and their diverse roles in biological steps, and emerging findings have revealed crucial functions of ncRNAs in transcriptional regulation. In addition, advances in genome-wide association studies (GWAS) have led to the discovery of new ncRNAs. Among the ncRNAs, diverse biological functions of lncRNAs have been identified, including in tumor biology, such as modulation of transcription and translation by acting as transcription factors, splicing of mRNA, modification of chromatin, interaction of ribosomes, sponging with miRNA, degradation of mRNAs, scaffolding of protein interactions, and trafficking of transcription factors [172]. Recently, several remarkable studies reported the relation of lncRNAs and EVs, which translocate the DNA, RNA, proteins, and lipids to other cells, resulting in diverse phenotypic responses that promote functional modulation of cellular activity in recipient cells [173, 174]. Furthermore, lncRNAs and EVs are closely related with various diseases. In addition, they are associated with tumorigenesis and tumor microenvironment [175–177]. Consequently, these unique functions and biology of the EVs in cell-to-cell communications through the delivery of cargo, including mRNAs, miRNAs, and lncRNAs, make them attractive as potential therapeutics and biomarkers in various diseases [178, 179].

Molecular insights into lncRNAs-mediated intracellular pathways and transcriptional networks have provided answers for several unanswered questions in transcriptional and translational regulation. Recent observations have also revealed the roles of lncRNAs in diverse diseases and responses to various stresses. Besides, abnormal transcription and mutation of lncRNAs have been observed in many sporadic tumors [180]. Indeed, while activation or inactivation of lncRNAs might affect tumor development, cancer cells may also rely on the molecular environment. Given the potential importance of lncRNAs in brain tumors, they are an ideal target for oligonucleotide-based therapeutics. However, several biological issues must be resolved before their successful application as the therapeutic agents. For example, a more rigorous approach for clinical evaluation of lncRNAs for brain tumor therapeutics requires the assessment of blood–brain barrier (BBB) permeability or stability in cerebrospinal fluid exposure [181]. Although circulating lncRNAs are derived from several sources including blood and serum, identification of the lncRNAs in mammalian fluids has been hampered by a lack of genetic information. Moreover, most studies have been considered and preferred to identify miRNAs present in the fluids in diseased state as therapeutic targets compared to lncRNAs in whole blood [182]. Previously, cell-free nucleic acids, such as DNA, mRNA, and miRNA were considered as biomarkers in cancer patients [183]. Recent advances in lncRNA research, which were based on GWAS and biochemical systems, have improved the knowledge of cell-free nucleic acids including circulating lncRNAs [184, 185]. This impressively wide range of findings can facilitate the establishment of preclinical models for identification of potent lncRNAs for application in clinical treatment in cancer [186]. Thus, it is worth studying specific interactions between lncRNAs and brain tumors, which may enable determination of lncRNA-targeted therapeutic strategies that might be used increasingly in the future.

In this review, we have summarized the current understanding of the roles of lncRNAs in brain tumors (Table 1). Understanding the various functions of gene transcripts has led to advancements in identification of biomarkers and drug development. We have reviewed recent insights into biological functions of lncRNAs that were gleaned through genetic and biochemical studies, though many unanswered questions remain. For example, the genetic or epigenetic mechanisms that regulate the expression, localization, and activity of lncRNAs are still unclear. In addition, a lack of certain transcription factors in lncRNA pathways raises the question of whether bidirectional mechanisms other than genetic functions of sense or antisense transcripts exist. Besides, it is not clear whether lncRNAs are fundamental molecules in brain tumor progression. The development of brain tumor involves several complex mechanisms regulated by multiple molecules and pathways. Cells are constantly challenged by diverse abnormalities caused by exogenous and endogenous factors. In addition, genomic instability caused by various factors is related to tumor evolution and patient outcomes [187]. In general, tumors are ascribed to genetic heterogeneity resulting from changes in processes, such as transcriptional or epigenetic regulation [188]. The biological features of lncRNAs are heterogenous; they are associated with *cis*- or *trans*-acting transcriptional regulation and they are involved in modulation of targeted RNAs or proteins [6]. Since the discovery of the biological roles of lncRNAs, there have been significant advances in the field of tumorigenesis [189]. Therefore, novel therapeutic approaches might also include targeting active lncRNAs that are essential for cell death or survival in brain tumors. High-throughput screening studies across several decades have focused on identifying non-coding RNAs that could serve as genomic signatures in biological systems [190]. Using this approach, the importance of lncRNAs as therapeutic targets has emerged in various cancers [189]. The diverse roles of lncRNAs in tumorigenesis could shed greater light on other undiscovered functions of lncRNAs and potentially provide new therapeutic approaches for treating brain tumors.

**Table 1:** Signaling pathway and role of lncRNA-target genes in brain tumors

lncRNA	Target	Signaling pathway	Activity	Reference
PVT1	miR-128-3p	BMP pathway	Promote tumorigenesis and progression	[91]
	miR-186		Promote cell proliferation, migration, and angiogenesis	[86]
	miR-200a		Promote cell proliferation and invasion	[87]
	miR-424		Promote cell viability, migration invasion and tumor growth	[88]
	miR-140-5p		Promote cell proliferation, invasion, and aerobic glycolysis	[89]
	miR-190a-5p/MEF2C		Promote malignant behaviors	[90]
	miR-488-3p/MEF2C			
CASC2	miR-181a	PTEN and Akt pathway	Inhibit cell growth and resistance to TMZ	[27]
	miR-193a-5p	mTOR pathway	Promote TMZ cytotoxicity through autophagy inhibition	[28]
		Wnt/β-catenin pathway	Inhibit cell proliferation, migration, and invasion	[62]
	miR-21		Inhibit cell proliferation, migration and invasion and promote apoptosis	[92]
ZFAS1		Notch pathway	Promote progression via regulating EMT	[96]
	miR-150-5p		Promote progression	[94]
	miR-432-5p		Promote viability and promote cisplatin cytotoxicity	[95]
DANCR		Wnt/β-catenin pathway	Promote progression through activation EMT	[63]
	miR-33a-5p miR-33b-5p miR-1-3p miR-206 miR-613a	PI3K/Akt/NF-κB pathway	Promote cisplatin resistance in vitro and in vivo	[34]
	miR-634/RAB1A		Promote progression	[97]
	miR-135a-5p/BMI1		Promote proliferation, migration, and invasion	[99]
HULC	ESM-1	PI3K/Akt/mTOR pathway	Promote angiogenesis by regulated ESM-1	[36]
SNHG16	miR-4518/PRMT5	PI3K/Akt pathway	Inhibit apoptosis and promote tumorigenesis via upregulating PRMT5	[37]
	miR-373/EGFR	PI3K/Akt pathway	Promote tumorigenesis via releasing EGFR	[38]
	miR-20a-5p/E2F1		Promote malignancy through increase E2F1	[100]
CRNDE	miR-136-5p/Bcl-2, Wnt2	PI3K/Akt/mTOR pathway	Promote malignancy via inhibiting Bcl-2 and Wnt2	[41]
	P70S6K	mTOR pathway	Promote cell growth and invasion via increasing phosphorylation of P70S6K	[42]
	miR-348/PIWIL4		Promote malignant progression via enhancing PIWIL4	[101]
	miR-186		Promote cell proliferation, migration, invasion, and tumor growth by inhibiting apoptosis	[102]
HOTAIR	miR326/FGF1	PI3K/Akt and MEK1/2 pathway	Promote apoptosis and cell cycle via inhibiting FGF1	[45]
	PRC2		Promote cell cycle progression via interacting PRC2	[103]
	VEGFA		Promote angiogenesis via increasing VEGFA	[104]
	miR-126-5p/GLS		Promote progression via regulating GLS	[105]
	miR-148-3p		Inhibit malignant biological behaviors	[106]
	miR-141/SKA2		Promote progression and tumor growth	[107]
	miR-148-3p/USF1		Inhibit BTB permeability via promoting USF1	[108]
	BRD4		Promote proliferation by regulation of BRD4	[109]
H19		NF-κB pathway	Promote TMZ resistance	[117]
		Wnt/β-catenin pathway	Promote proliferation, migration, and invasion	[69]
		Wnt/β-catenin pathway	Promote TMZ resistance through suppressed EMT	[70]
	miR-152		Promote proliferation and invasion	[110]
	miR-140/iASPP		Promote proliferation and invasion by targeting iASPP	[111]
	miR-675		Promote proliferation, migration invasion and tumor growth	[66, 68, 112, 113]
	miR-29a/VASH2		Promote angiogenesis	[114]
	miR-138/HIF-1α		Promote proliferation, migration, invasion, and angiogenesis by targeting HIF-1α	[115]
	miR-130a-3p/SOX4		Promote migration, invasion and EMT via regulating SOX4	[116]
XIST	miR-126/IRS1	PI3K/Akt pathway	Promote cell growth, glucose metabolism and tumor growth via regulating IRS1	[48]
	miR-429		Promote angiogenesis and tumorigenicity	[118]
	miR-29c/SP1/MGMT		Promote proliferation and TMZ resistance through the DNA mismatch repair pathway	[119]
	miR-137/Rac1		Promote progression by upregulated Rac1	[120]
	miR-137/FOXC1/ZO-2		Inhibit BTB permeability and promote angiogenesis by increasing FOXC1 and ZO-2	[121]
	miR-152		Promote progression and decrease apoptosis	[122]
NEAT1	EGFR	Wnt/β-catenin pathway	Promote progression and tumorigenesis through EGFR	[76]
	miR-449b-5p/c-Met		Promote pathogenesis by upregulated c-Met	[123]
	miR-let-7e/NRAS		Promote proliferation migration and invasion while inhibit apoptosis by upregulated NRAS	[124]
	miR-132/SOX2		Promote migration and invasion through upregulation of SOX2	[125]
	miR-107/CDK6, 14		Promote malignant progression by promoted CDK6, 14	[126, 127]
	miR-181-5p/SOX5		Inhibit BTB permeability via upregulating SOX5	[128]
MALAT1	WIF1	Non-canonical Wnt pathway	Promote migration via regulating of WIF1	[77]
		ERK/MAPK pathway	Inhibit proliferation and invasion	[55, 56]
	miR-199a/ZHX1		Promote proliferation and progression by increase ZHX1	[129]
	miR-129/SOX2		Promote tumorigenesis via facilitating of SOX2	[130]
	miR-101		Promote progression and TMZ resistance	[131–133]
	miR-203		Promote TMZ resistance	[134]
	miR-155/FBXW7		Inhibit cell viability by upregulated FBXW7	[135]
MATN1-AS1	RELA	MAPK pathway	Inhibit cell proliferation and invasion through the apoptosis	[57]
	miR-200b/c/429/CHD1		Promote glioma progression	[136]
TPTEP1	miR-106a-5p	P38 MAPK pathway	Inhibit stemness and radio resistance	[59]
CCAT2	miR-424/VEGFA	PI3K/Akt pathway	Promote proliferation and angiogenesis via increasing VEGFA	[50]
		Wnt/β-catenin pathway	Promote proliferation, migration, cell cycle and tumorigenesis	[78]
	miR-424/Chk1		Promote chemodrug resistance by upregulated Chk1	[137]
			Promote angiogenesis and inhibit endothelial cell apoptosis through release CCAT2-enriched exosomes	[169]
PGM5-AS1	risk-related genes		Regulate several prognostic immune-related mRNA	[147]
ST20-AS1				
AGAP2-AS1				
MIR155HG				
SNHG8	protective genes		Regulate several prognostic immune-related mRNA	
LINC00937				
TUGI				
MAPKAPK5-AS1				
HCG18				
AK098425	protein coding gene		Associate immune-related biological immunity	[150]
AL833059				
AK056155				
CR613436				
AC005013.5	protein coding gene		Associate immune-related biological process and pathway	[151]
UBE2R2-AS1				
RP11-89C21.2				
AC073115.6				
XLOC-004803				
HOTAIRM1	miR-129-5p		Promote malignancy and relate immune activation	[152]
POU3F3			Promote angiogenesis and release POU3F3-enriched exosomes	[165]
SBF2-AS1	miR-151a-3p		Promote TMZ resistance and secret SBF2-AS1-enriched exosomes	[168]
ATB	miR-204-3p		Promote cell activation and invasion through release ATB-enriched exosomes	[171]

## Data Availability

Not applicable.

## Abbreviations

ATB: Activated by transforming growth factor-β
Bcl-2: B-cell lymphoma 2 family
Bax: Bcl-2-associated X
BTB: Blood–tumor barrier
BBB: Blood–brain barrier
BMI1: B-lymphoma Moloney murine leukemia virus insertion region-1
BMP: Bone morphogenetic protein
BRD4: Bromodomain-containing 4
CASC2: Cancer susceptibility candidate 2
CCAT2: Cancer susceptibility candidate 2
CHD1: Chromodomain helicase DNA-binding protein 1
CRNDE: Colorectal neoplasia differentially expressed
ceRNAs: Competing endogenous RNAs
CDK6: Cyclin-dependent kinase 6
DANCR: Differentiation antagonizing non-protein coding RNA
ESM-1: Endothelial cell-specific molecule 1
EZH2: Enhancer of zeste 2
EGFR: Epidermal growth factor receptor
EMT: Epithelial–mesenchymal transition
ERK: Extracellular signal-regulated kinase
EVs: Extracellular vesicles
FBXW7: F-box and WD repeat domain-containing 7
FGF1: Fibroblast growth factor 1
FOXC1: Forkhead box C1
GWAS: Genome-wide association studies
GREM1: Gremlin 1
HULC: Highly upregulated in liver cancer
HOTAIR: HOX transcript antisense RNA
IGF2: Insulin growth factor 2
IRS1: Insulin receptor substrate 1
JAG1: Jagged 1
LncRNAs: Long non-coding RNAs
MATN-AS1: MATN1 antisense RNA 1
MMP2: Matrix metalloproteinase 2
MALAT1: Metastasis-associated lung adenocarcinoma transcript 1
miRNAs: MicroRNAs
MAPKs: Mitogen-activated protein kinases
MEF2C: Myocyte enhancer factor 2C
NF-κB: Nuclear factor kappa-light-chain-enhancer of activation B cells
NEAT1: Nuclear-enriched abundant transcript 1
MGMT: O6-methylguanine-DNA methyltransferase
PTEN: Phosphatase and tensin homolog
PIWIL4: Piwi-like RNA-mediated gene silencing 4
PVT1: Plasmacytoma variant translocation 1
PRC2: Polycomb repressive complex 2
POU3F3: POU class 3 homeobox 3
PRMT5: Protein arginine methyltransferase 5
PLP2: Proteolipid protein 2
Rac1: Ras-related C3 botulinum toxin substrate 1
SBF2-AS1: SBF2 antisense RNA 1
SOX2: Sex determining region Y-box protein 2
SOX4: Sex determining region Y-box protein 4
SOX5: Sex determining region Y-box protein 5
SNHG16: Small nucleolar RNA host gene 16
SP1: Specificity protein 1
SKA2: Spindle and kinetochore associated complex subunit 2
TMZ: Temozolomide
TGF-β: Transforming growth factor-β
TPTEP1: Transmembrane phosphatase with tensin homology pseudogene 1
USF1: Upstream stimulatory factor 1
VEGFA: Vascular endothelial growth factor A
VASH2: Vasohibin 2
WIF1: Wnt inhibitory factor 1
XIST: X-inactive-specific transcript;
ZHX1: Zinc fingers and homeoboxes 1
ZFAS1: ZNFX1 antisense RNA 1
ZO-1: Zonula occludens 1
